# The Chorioallantoic Membrane Assay in Nanotoxicological Research—An Alternative for *In Vivo* Experimentation

**DOI:** 10.3390/nano10122328

**Published:** 2020-11-24

**Authors:** Christoph R. Buhr, Nadine Wiesmann, Rachel C. Tanner, Jürgen Brieger, Jonas Eckrich

**Affiliations:** 1 Department of Otorhinolaryngology, University Medical Center of the Johannes Gutenberg-University Mainz, Langenbeckstraße 1, 55131 Mainz, Rhineland-Palatinate, Germany; r.ch.buhr@gmail.com (C.R.B.); nwiesman@uni-mainz.de (N.W.); racheltanner@web.de (R.C.T.); Jonas.eckrich@unimedizin-mainz.de (J.E.); 2 Department of Oral and Maxillofacial Surgery, -Plastic Surgery, University Medical Center of the Johannes Gutenberg-University Mainz, Langenbeckstraße 1, 55131 Mainz, Rhineland-Palatinate, Germany

**Keywords:** nanoparticles, toxicology, animal models, *in vivo* models, rodent models, CAM assay, CAM model, chorioallantoic membrane assay, nanotoxicology

## Abstract

Nanomaterials unveil many applicational possibilities for technical and medical purposes, which range from imaging techniques to the use as drug carriers. Prior to any human application, analysis of undesired effects and characterization of their toxicological profile is mandatory. To address this topic, animal models, and rodent models in particular, are most frequently used. However, as the reproducibility and transferability to the human organism of animal experimental data is increasingly questioned and the awareness of animal welfare in society increases at the same time, methodological alternatives are urgently required. The chorioallantoic membrane (CAM) assay is an increasingly popular *in ovo* experimental organism suitable for replacement of rodent experimentation. In this review, we outline several application fields for the CAM assay in the field of nanotoxicology. Furthermore, analytical methods applicable with this model were evaluated in detail. We further discuss ethical, financial, and bureaucratic aspects and benchmark the assay with other established *in vivo* models such as rodents.

## 1. Introduction

Nanomaterials have become an integral part of everyday life in the 21st century, which is reflected in an growing number of consumer products containing nanomaterials and an increasing number of publications dealing with nanotechnology [1]. Nanomaterials have also evoked an increasing popularity for versatile medical applications, which range from imaging techniques [2,3,4] to the use as drug carriers [5].

However, biocompatibility of nanoparticles is still not fully understood and remains the subject of current research [6]. More specifically, toxicological profiles (lethality, application ways, circulation kinetics or biodistribution) of nanoparticulate substances for many types of nanoparticles have not been elucidated yet. According to Graham et al. 2019 “the dose response relationship is complicated by the physicochemical transformations in the nanoparticles induced by the biological system producing an altered response” [6].

Due to their small size nanoparticles are able to enter organ structures which are usually not exposed to bulk materials [7]. Bulk materials include bigger particles of the same material exceeding the nano range [8,9]. Nanoparticles have deviant pharmacokinetic profiles compared to native bulk materials [6,7]. As a decrease in nanoparticulate size results in a relative increase of their surface, the number of reactive groups consecutively rises as well, resulting in a higher overall reactivity [7]. Hence an inverse relationship between size and toxicity of nanoparticles is discussed [7,10]. Furthermore, the shape of nanoparticles can also influence their toxicity and toxicokinetic [11,12].

Since biocompatibility and toxicity of nanoparticles are pending questions [6], further research is urgently needed to understand the mechanisms of nanoparticle-cell interaction within the organism. Animal models provide insights into the interactions between nanoparticles and living creatures and have been established in the field of toxicology since centuries [13,14].

However, even though animal models allow quantification and evaluation of toxicity, concerns regarding the ethical justifiability of animal research emerge [13,14]. Today animal research faces a lot of controversy due to an increasing awareness for animal welfare in society [13]. The “3R principle” (reduce, refine, replace) has been established as a benchmark in scientific research today [15]. Accordingly, the use of animal models must be carefully addressed. Taking these challenges into consideration, hens eggs models have become increasingly popular for toxicological experiments in recent years (Table 1). An extended version of this table, containing more detailed information on the nanomaterials used by the authors, is provided in the (Supplementary Materials Table S1).

**Figure 1:**
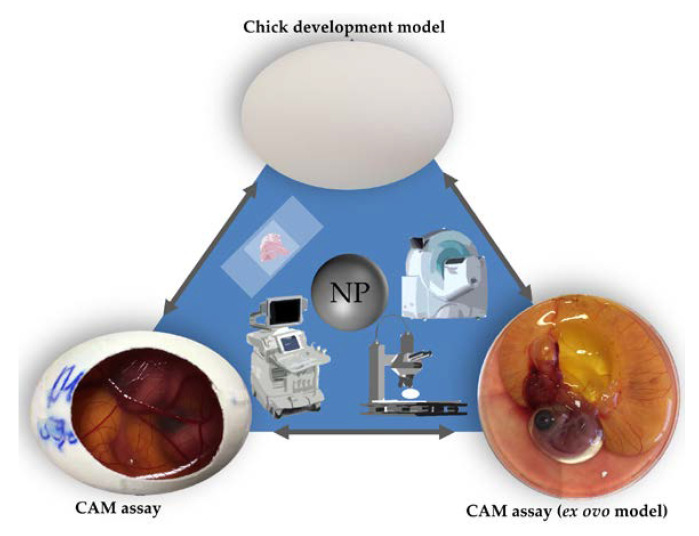
Three main *in ovo* techniques with associated imaging methods.

The chorioallantoic membrane (CAM) assay in a non-innervated, highly vascularized, extraembryonic membrane [57,58]. The CAM can be considered to be placental equivalent to mammals and has been established itself as a versatile alternative to conventional rodent experimentation and meets the criteria of the 3R principle [57,58,59,60]. As chicken embryos are living vertebrates with a circulatory system and organic functions, this model can serve as a suitable substitute for various *in vivo* experimentation (Figure 1) [57,60,61]. Since Luepke identified the CAM assay as an alternative *in vivo* method to evaluate mucosa irritation [61], the assay has substituted the Draize Eye Irritation Test for various substances [62,63,64,65]. Further evaluation of the CAM assay might illustrate the suitability of this model for versatile applications and translate to an increased use in toxicological research.

In this review, we discuss the eligibility of the CAM assay as an alternative for rodent experiments in the field of nanotoxicological research.

More specifically, we outline several application fields for the CAM assay in the field of nanotoxicology. Furthermore, analytical methods applicable with this model were outlined in detail.

## 2. Review Criteria

Table 1 summarizes the results of a systematic database search in Medline (https://pubmed.ncbi.nlm.nih.gov) with the following search commands: “((CAM assay) OR (CAM model) OR (chorioallantoic membrane assay) OR (chorioallantoic membrane model) OR (*in ovo*) OR (*ex ovo*)) AND ((nanoparticle) OR (nanoparticulate) OR (nanoparticular)) AND ((toxicity) OR (toxic effects) OR (toxicology))”. The definition of “nanoparticle” is a controversially discussed topic [9]. Since this review focuses on methodology rather than the nanoparticulate entity, no further specifications regarding the types of nanoparticles used in the specific study were applied. Following our selection criteria, only publications dealing with the CAM assay were included in the table. Experimental setups using the CAM assay without any toxicological questioning were excluded. A division into *in ovo* and *ex ovo* methods was made. When authors did not mention a special setup an *in ovo* method was assumed. A Prisma 2009 flow diagram, based on our review criteria, can be found in the (Supplementary Materials Figure S1).

## 3. The Evaluation of Nanotoxicity with Fertilized Hen’s Eggs

Two models making use of fertilized hen’s eggs have been established as the main tools in toxicological research: the chicken development model and the CAM assay (Figure 1) [16,17,18,19,20,21,22,23,24,25,26,27,28,29,30,31,32,33,34,35,36,37,38,39,40,41,42,43,44,45,46,47,48,49,50,51,52,53,54,56,66,67,68,69,70,71,72,73,74,75]. The chicken development model evaluates toxicological effects of specific substances on embryogenesis without further manipulation of the egg [66,67,68,69,70,71,72,73,74,75]. In this model, a specific substance is administered into the egg. After a defined period of time the experiment is terminated by opening the egg allowing a variety of end point analytical methods [66,67,68,69,70,71,72,73,74,75].

**Figure 2:**
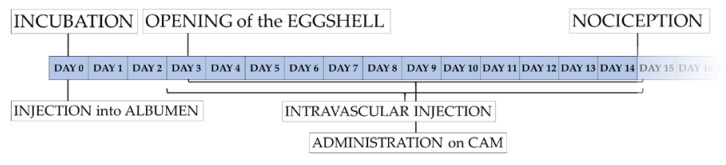
Timeline of experimentation with the CAM assay *in ovo* method.

In the CAM assay, the chorioallantoic membrane is exposed by partial removal of the eggshell. Thus, the embryonic development can be longitudinally observed though the opening (Figure 2) [60]. The highly vascularized CAM can further serve as an analytical platform evaluating chemical irritation [16,17,18,19,20,21,22,23,24,25,26,27,28,29,30,31]. Therefore the CAM assay has already been used for various experimental setups including angiogenesis [76], wound healing [77], tumor development [78], and specifically toxicological research [79,80,81,82].

Due to its increasing popularity and the variety of possible experimental setups, in this review we will focus on the CAM assay.

### 3.1. Experimental Setup of the CAM Assay—Ex Ovo vs. In Ovo Method

In general, the CAM assay can be performed as an *ex ovo* or *in ovo* method (Figure 1) [57]. In the *ex ovo* models after breaking the eggshell the eggs content is transferred to an alternative container for example a petri dish, or a cup [83]. Here further development can be observed without impairment of insight by the eggshell. Accessibility of the CAM surface as well as an increased visibility can be considered to be the key advantages of the *ex ovo* model over the *in ovo* model [57]. However, the transfer of the chicken embryo and the CAM leads to increased dropout rates, which can be considered to be the main disadvantage of the *ex ovo* model [57].

When using the *in ovo* method, the eggshell is only partially removed [57]. Once opened the scientist has direct access to the developing CAM. In contrast to the *ex ovo* method only a small sector of the CAM becomes visible and can be manipulated (Figure 1) [57]. The main advantage of the *in ovo* model is a more simple setup as well as much lower dropout rates, in comparison to the *ex ovo* model [57].

### 3.2. Application Methods for Nanoparticulate Substances

Different application methods have influence on nanoparticulate toxicity [84]. The CAM assay allows various applicational modalities: Injection into the albumen [66,67,68,69,70,71,72,73,74,75], direct admission onto the CAM surface [16,17,18,19,20,21,22,23,24,25,26,27,28,29,30,32,33,34,35,36,37,38,39,40,41,42,43,45,46,47,48,52,55,56], intravascular injection [31,49,50,51,56], and intracardiac application [54] have been described for evaluation of nanoparticle toxicity.

In terms of direct admission onto the CAM surface, some authors placed the nanoparticles inside a ring of plastic [42], silicone [33] or Teflon^®^ [39,52], while others applied the nanoparticles to the CAM using Delrin^®^ containers [47], (soaked) filter paper or disks [32,35,36,38,40,41].

### 3.3. Analytical Methodologies Provided by the CAM Assay 

Free access to the CAM allows a variety of analytical methods (Figure 1) [57]. While some researchers evaluate chemical irritation of a specific substance on the CAM [16,17,18,19,20,21,22,23,24,25,26,27,28,29,30,31], further investigation such as vascularization or developmental changes can also be addressed [32,33,34,35,36,37,38,39,40,41,42,43,44,45].

Since the opening of the eggshell allows direct observation of the CAM optical evaluation by microscopy [29,31,32,33,36,37,42,43,44,45,46,47,48,49,50,52] as well as imaging techniques such as ultrasonography [85], computed tomography [86], and magnetic resonance tomography [87] have been successfully used in the CAM assay (Figure 1).

Regarding overall toxicity the median lethal dosage (LD_50_) can also be determined using this specific methodology [82].

#### 3.3.1. Methods to Assess Vascularization

The investigation of angiogenesis is a frequent use of the CAM assay [57]. As the CAM has a dense vascular network extending during development, the impact of nanoparticles on angiogenesis and vasculogenesis can be observed in detail [58]. Yet, several authors have evaluated the influence of different nanoparticulate substances on angiogenesis [32,33,34,35,36,37,38,39,40,41,42,43,44,45]. The number of vessel branches as well as vessel size and vascular density are frequently used as parameters for quantification [34,37,40,41,43,44,45]. A detailed methodological description of the monitoring of microvessel density as well as vessel branches and junctions was recently published by Heimes et al. [88]. Histological and immunohistochemical analysis of the CAM can also be used for the determination of vessel density [42]. More specifically, clotting factor 8 [42], lectins [89,90,91,92], CD-31 [93], desmin [93], and anti-smooth muscle antigen (alpha-SMA) [60] are frequently used to visualize vessels in histological specimens. While the use of lectins from Lens culinaris agglutinin (LCA) [89,90,91], and from Sambucus nigra (SNA) [90,92] represent more established methods, the use of CD-31 is controversial in the CAM [91]. Other authors used surrogate parameters such as messenger RNA (mRNA) expression of different proangiogenic factors such as vascular endothelial growth factor (VEGF), vascular endothelial growth factor receptor 2 (VEGFR2), or fibroblast growth factor (FGF) [38].

#### 3.3.2. Methods to Assess CAM Damage and Irritation

As mentioned above the CAM assay substituted the painful Draize Eye Test for the evaluation of mucosal irritation potential [61]. To objectively monitor irritation of the CAM a “irritation score” (IS) was created, which is based solely on visual observation. This score, which is used by most authors to quantify irritation, is determined by the occurrence of lysis (L), hemorrhage (H), or coagulation (C) in relation to a specific time point after application. A value between 0 and 21 is calculated afterwards (IS = (301 − H) × 5/300 + (301 − L) × 7/300 + (301 − C) × 9/300), whereas 0.0–0.9 equals no irritation; 1.0–4.9 equals slight irritation; 5.0–8.9 equals moderate irritation and 9.0–21.0 equals serve irritation [17,18,21,23,25,26,30].

#### 3.3.3. Methods to Assess *In Vivo* Circulation

Characterization of toxicokinetics is a prerequisite for establishing nanoparticles as potential drug-carriers in clinical practice [84]. Therefore, a better understanding of intravascular *in vivo* behavior and circulation are necessary. With its highly vascularized membrane the CAM assay offers unique conditions for the evaluation of circulatory profiles. Different authors monitored intravascular particle movement and particle distribution using fluorescent dye labeling [51,94,95,96].

Whereas Vu et al. 2018 used PMO (periodic mesoporous organosilica) nanoparticles loaded with doxorubicin [51] and Cho et al. 2011 used nanoparticles derived from Cowpea mosaic virus (CPMV) conjugated with Alexa Fluor 647 [96], Smith et al. 2011 used “indirect visualization by co-injecting the plasma marker FITC dextran [94].

#### 3.3.4. Methods to Determine the Median Lethal Dosage (LD_50_)

The median lethal dosage (LD_50_) has been established as a standard value for the quantification of toxicity [97]. So far LD_50_ calculation of nanoparticles using the CAM assay has not yet been described. Although some authors reported survival rates after nanoparticle application, a systematic quantification has not been established yet. Kue et al. have determined the LD_50_ of various chemotherapeutic pharmaceuticals by using the CAM assay [82].

#### 3.3.5. Further Analytical Methods *In Ovo*

In addition to the techniques mentioned above, further methodology *in ovo* has been established in the chicken development model [71,74,98]. As described in the following, for many of these techniques, due to the methodological similarities, equivalent evaluations in the CAM assay would also be possible; however, it has not yet been described in the literature.

Prasek et al. and Sikorska et al. for example described blood analysis *in ovo* evaluating levels of alanine aminotransferase (ALT), aspartate aminotransferase (AST), lactate dehydrogenase (LDH), alcaic phosphatase (ALP), glucose level, or urea levels [71,74]. Moreover, organ damage determined by immunohistochemistry monitoring cellular proliferation (Proliferating-Cell-Nuclear-Antigen (PCNA) [71,74,98]; or apoptosis (Caspase-3) [74] has also been described *in ovo*. Other authors used BromodeoxyUridine (BrdU) [58,92] as an alternative marker for cellular proliferation in the CAM; however, to our knowledge BrdU has not yet been used to monitor organ damage in the CAM assay. 

Further analytical methods such as *in ovo* imaging with computer tomography [86], magnetic resonance tomography [87] or ultrasound [85] have already been established in combination with the CAM assay, yet not for scientific questions in the field of nanotoxicology. Monitoring of organ damage with the techniques mentioned above for example would be a way of evaluating nanotoxicological effects.

### 3.4. General Procedure of the CAM Assay Performed as In Ovo Method

**Figure 3:**
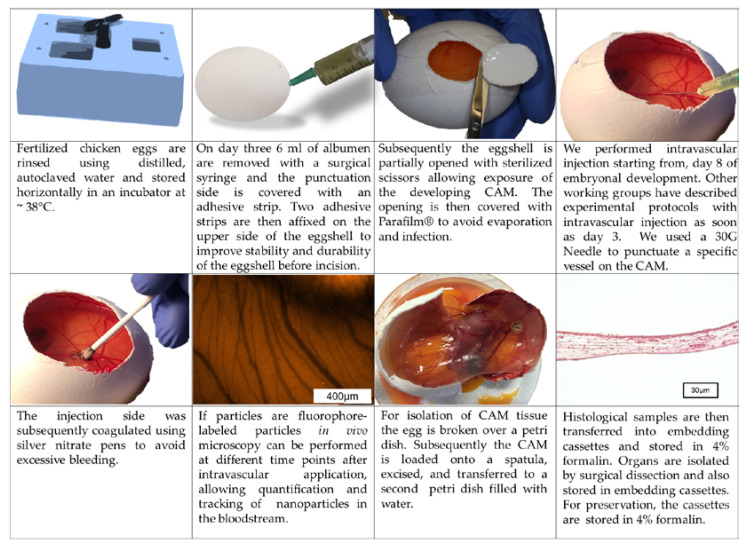
Technique of the CAM assay (*in ovo* model) as established in our laboratory.

Although time points for manipulation and the detailed procedure of the CAM assay may vary between different working groups, the principal methodology of the CAM assay is rather similar between different protocols [16,17,18,19,20,21,22,23,24,25,26,27,28,29,30,31,32,33,34,35,36,37,38,39,40,41,42,43,44,45,46,47,48,49,50,51,52,53,54,55,56]. Here we will describe the methodological procedure that is used in our working group (Figure 2 and Figure 3) [60]:

Fertilized chicken eggs are rinsed using distilled, autoclaved water and stored horizontally in an incubator at ~38 °C (Figure 2 and Figure 3). After 3 days of incubation 6 ml of albumen are removed with a surgical syringe and the punctuation side is covered with an adhesive strip (Tesafilm^®^, Tesa SE, Hamburg, Germany) (Figure 2 and Figure 3). Two adhesive strips (Leukosilk^®^, BSN Medical, Hamburg, Germany) are then affixed on the upper side of the eggshell to improve stability and durability of the eggshell before incision. Subsequently the eggshell is partially opened with sterilized scissors allowing exposure of the developing CAM (Figure 2 and Figure 3). The opening is then covered with Parafilm^®^ (Bemis Company Inc., Neenah, WI, USA) to avoid evaporation and infection. The application of substances for toxicological evaluation can then be performed on different points in time. Injection into the albumen can be performed starting from day 0, whereas administration onto the CAM surface is obviously only possible after shell removal (Figure 2 and Figure 3). Due to very small vessel diameters intravascular injections are very hard to perform before day 8 of embryo development in our experience; however, other working groups have described experimental protocols with intravascular injection as soon as day 3 [50]. With the increasing age of the eggs, however, the injection of substances into the blood vessel system becomes easier. After the injection, the bleeding can be coagulated using silver nitrate pens to avoid excessive bleeding (Figure 3).

Histological and immunohistochemical analysis require formalin fixation and thus can only be performed as endpoint measurements. For isolation of CAM tissue the egg is broken over a petri dish (Figure 3). Subsequently the CAM is loaded onto a spatula, excised, and transferred to a petri dish filled with water, while floating in the water the plane mounting of the CAM on a filter paper will be much easier to perform. Afterwards filter paper with the CAM is wrapped into embedding cassettes (Carl Roth GmbH und Co. KG, Karlsruhe, Germany) and transferred in 4% formalin (VWR International bvba, Leuven, Belgium). Organs of the embryo are isolated by surgical dissection and also stored in embedding cassettes to be transferred into 4% formalin.

### 3.5. The CAM Assay for Evaluation of Nanotoxicity—Current Status

Our recherche yielded 75 hits, 15 results included neither the CAM assay nor the chick development model. Further 10 results addressed the chick development model, whereas 9 results used the CAM assay but did not address a toxicological question. The remaining 41 publications are shown in Table 1. Only six publications described an *ex ovo* setting as methodological entity. Interestingly, one author described the use of turkey eggs instead of hen’s eggs [55]. 

The “Irritation Test”, as exemplarily described above, performed on the CAM assay was the methodology most frequently used for toxicological evaluation of nanoparticles within our search (16/41 publications) [16,17,18,19,20,21,22,23,24,25,26,27,28,29,30,31]. The evaluation of angiogenesis, as described earlier, was also a recurrent observational focus (13/41 publications) [32,33,34,35,36,37,38,39,40,41,42,43,44,45]. Further working groups evaluated morphological changes of the embryo after nanoparticulate application of zinc oxide nanoparticles [43], titanium dioxide nanoparticles [52] or liposome encapsulated dendriplex systems [53]. Other authors monitored more special parameters like femur ossification [44].

Most authors performed administration of the investigated substances via deposition of the nanoparticles onto the CAM surface (33/41 publications) [16,17,18,19,20,21,22,23,24,25,26,27,28,29,30,32,33,34,35,36,37,38,39,40,41,42,43,45,46,47,48,52,55]. Whereas four authors described intravascular application [31,49,50,51,56], special application methods such as injection into the heart [54] or injection into the mesoderm were rarely described [53].

### 3.6. Ethical, Financial, and Bureaucratic Aspects of the CAM Assay

The CAM assay is considered to suffuse higher ethical standards when compared to other animal models [91]. From an ethical standpoint lack of nociceptive nerves in the CAM as well as the chick embryos absence of nociception till 14 due to unfinished neuronal differentiation [57] make the model a favorable alternative compared to equivalent rodent models.

An established benchmark for scientific ethical standards is represented by the “3R principle” (reduce, refine, replace) [15]. The CAM assay meets these criteria for two reasons. First the CAM assay refines animal models through a direct access to the CAM without any surgical intervention on the organism itself. In contrast to the surgical exposure of the vessel network in rodents, such as the dorsal skinfold chamber [99] or cremaster muscle imaging [100] the CAM is exposed without any incisions, thereby eliminating the specific effect of the surgical intervention. In the field of nanotoxicological research it can be applied for *in vivo* analysis of circulating nanoparticles [51,94,95,96] or evaluate their impact on vascularization [32,33,34,35,36,37,38,39,40,41,42,43,44,45]. In addition, the CAM assay is not considered to be an animal model in most developed countries [57,101], consequently the assay fulfils the criteria of replacement. However, there are still ethical concerns as the embryo develops nociception from day 14 on [57]. Accordingly, in most published experimental setups experimentation is terminated before day 14 of development [16,17,18,19,20,21,22,23,24,25,26,27,28,29,30,31,33,34,35,36,37,38,39,40,41,42,43,46,47,48,49,50,52,54].

The CAM assays status as an *in vivo* model without being an animal model per definition results in way lower bureaucratic hurdles when compared to rodent models [57,101]. No ethical approval is needed for experimentation with the CAM assay in most countries [57,101]. Beyond that in most institutions no license for care and experimentation with this model must be obtained. Hence it allows *in vivo* experimentation in laboratories which do not have a license for animal care and conduction of animal experiments. As recently discussed by our working group the cost factor can further be considered a key advantage of this model [60]. Fertilized chicken eggs carry a far lower financial burden then mice. Furthermore, the costs for rodents, due to the requirement of food, housing, and personnel, far exceed running costs of the CAM assay as well. These factors, in combination with a simple methodology allow a very high quantitative output which subsequently translates to a better standardization and reproducibility.

## 4. Discussion

Research on nanoparticle toxicity *in vivo* poses enormous challenges for scientists [6,84]. Apart from evaluation of dosage-dependent toxicity and differences in toxicity owing to different application techniques, differences in nanoparticle size and shape may result in an alternating toxicological profile [6,84]. Hence, for toxicological evaluation of nanoparticulate substances an extremely large number of animals would be needed to characterize a single substance [6,84]. This results in the urgent need for alternative methods allowing a cost-effective and well reproducible evaluation of nanoparticulate toxicity, which resembles the complex conditions which influence toxicity in organisms [6,84]. The CAM assay, due to its low cost and simple experimental setup might facilitate just that.

So far rodent models have been established as the methodological entity to address scientific questions regarding nanotoxicity [102]. Mice and rats in particular have become an integral part of toxicological research due to their relatively fast reproduction rate, low space requirements, and widespread availability [103]. Rodents are considered to be a representative organism with a good transferability of experimental findings owing to a physiology similar to humans [103]. In addition to overall toxicity, circulation patterns, and organ distribution of nanoparticles *in vivo* have been frequently investigated in rodent models as well using fluorescent or radioactive labeling in order to monitor accumulation of nanoparticles [104,105]. For instance, ZnO nanoparticles were evaluated using both fluorescence labeling (Cy5.5-NH) [105], as well as radioactive 18F-labeling *in vivo* [104].

Nanoparticles may enter the organism in different ways through exposition and application [106,107]. Whereas exposition includes unintended intrusion, application describes the intended entering of the human body [106,107]. Hence experimental setups need to reproduce these different application ways adequately.

Various applicational modalities including inhalation, oral, intravenous, intraperitoneal as well as subcutaneous admission are investigated and evaluated using rodent models [108].

Similarly, many application ways (injection into the albumen [66,67,68,69,70,71,72,73,74,75], intravenous injection [31,49,50,51,56], admission through the CAM surface etc. [16,17,18,19,20,21,22,23,24,25,26,27,28,29,30,32,33,34,35,36,37,38,39,40,41,42,43,45,46,47,48,52,56]) can be realized using the CAM assay as well. Beyond that, due to the exposed vascular network on the CAM *in vivo* circulation of nanoparticles can be monitored, without much effort if fluorophores are applied [51,94,95,96]. Furthermore, toxicokinetic in the form of organ distribution can be evaluated after termination of *in vivo* experimentation by dissection and histological analysis [70,71,74]. The ability to not only evaluate the circulatory kinetics of nanoparticles [51,94,95,96] but to further characterize their distribution in combination with the evaluation of dropout rates [51] and the calculation of toxicological reference parameters such as the LD_50_ [82] allows a multidirectional view on toxicological characteristics. As mentioned above, further analytical methods such as immunohistochemistry [71,74,98] or imaging techniques [85] allow the characterization of toxicological effects on specific organs.

Apart from discussed ethical aspects, low costs, lower bureaucratic obstacles, and the simple experimental setup are obvious advantages [60]. The uncomplex setup enables scientists to learn the methodology of the CAM assay rather quickly. In comparison to the complex models exposing the vascular network in rodents, the CAM assay does neither require surgical skills nor a complex operative setup. In addition, the time expenditure needed for experimentation with the CAM assay is low. Once the eggs are opened (*in ovo* method) or transferred to a petri dish (*ex ovo* method) no further interventions are required. Incubation of eggs does not consume any time for feeding or cleaning of cages resulting in a comparatively low workload. This low workload allows large experimental groups, which increases the reproducibility and standardization of *in vivo* experimentation.

However, one general limitation of the CAM assay is its short investigation period. Since the observational timeframe is either terminated by the hatching of the embryo on day 21 [109] or the discontinuation of experimentation on day 14 due to nociception [57], only short term toxicity is measurable. Another limitation is the fact that chickens are no mammals [58]. The translation of findings obtained *in ovo* to humans therefore bears a higher risk of insufficiency and might be considered unreliable. However, similar arguments can be stated for other established animal models as well. Oral and inhalative administration of nanoparticles are not possible using the CAM assay. 

Additionally, a chicken embryo is a rapidly growing organism with a high cellular proliferation rate and ongoing organogenesis [109]. Depending on the time of experimentation, organs might still exist in preliminary stages (e.g., pronephros, mesonephros, metanephros) [109] resulting in alternating metabolism and ultimately deviant toxicological profiles compared to fully developed organisms.

Furthermore, the lack of a specific immune system until day 14 does not allow the investigation of any immunomodulatory effects of a specific nanoparticulate substance thereby resulting in a possible misinterpretation of toxicity [110]. Therefore, the interpretation of the biocompatibility of nanomaterials might be impaired. Moreover, scientists must bear in mind that the CAM assay uses a developing embryo as an experimental platform. Some interpretational limitations may be facilitated by the fact that organs are not fully developed during experimentations [109] and therefore might react differently to the specific substance investigated. Finally, absence of egg specific antibodies for sale impairs the applicability of methods like immunohistochemistry. Increasing popularity of this model, however, might lead to an increased supply with these antibodies in the future.

## 5. Conclusions

Whereas rodent models require complex experimental conditions, the CAM assay provides a comparatively simple setup, which is still suited for even advanced scientific questions. However, further experiments evaluating the transferability of experimentation between different animal models and the CAM assay are urgently needed to allow standardization of the CAM assay in the field of toxicological research. 

Beyond some limitations the CAM assay offers a full-featured *in vivo* model, with low costs, less work effort, and low bureaucratic burdens. At the same time, the CAM assay meets higher ethical criteria (3R principle) when compared with rodent models. 

## Figures and Tables

**Table 1:** This table summarizes the results of a systematic database research in Medline (https://pubmed.ncbi.nlm.nih.gov) using the following search commands: “((CAM assay) OR (CAM model) OR (chorioallantoic membrane assay) OR (chorioallantoic membrane model) OR (*in ovo*) OR (*ex ovo*)) AND ((nanoparticle) OR (nanoparticulate) OR (nanoparticular)) AND ((toxicity) OR (toxic effects) OR (toxicology))”. The definition of “nanoparticle” is a controversially discussed topic. Since this review focuses on methodology rather than the nanoparticulate entity, no further specifications regarding the types of nanoparticles used in the specific study were applied. Following our selection criteria, only publications containing the CAM assay were included. Experimental setups using the CAM assay without any toxicological questioning were excluded. A division into *in ovo* and *ex ovo* methods was made. When authors did not mention a special setup an *in ovo* method was assumed.

Scientific Scope	Model	Application	Day of Application	Nanoparticle	Ref
Irritation Score	*in ovo*	CAM surface	9	polymeric NP	[16]
	*in ovo*	CAM surface	7	polymeric NP	[17]
	*in ovo*	CAM surface	10	polymeric NP	[18]
	*in ovo*	CAM surface	10	polymeric NP	[19]
	*in ovo*	CAM surface	10	polymeric NP	[20]
	*in ovo*	CAM surface	9	polymeric NP	[21]
	*in ovo*	CAM surface	10	polymeric NP	[22]
	*in ovo*	CAM surface	10	polymeric NP	[23]
	*in ovo*	CAM surface	8	lipid NP	[24]
	*in ovo*	CAM surface	10	lipid NP	[25]
	*in ovo*	CAM surface	10	lipid NP	[26]
	*in ovo*	CAM surface	9	lipid NP	[27]
	*in ovo*	CAM surface	9	lipid NP	[28]
	*in ovo*	CAM surface	9	lipid NP	[29]
	*in ovo*	CAM surface	9	polysaccharide nanobased nail formulation	[30]
Irritation Score, lethality	*ex ovo*	intravascular	4	polymer NP	[31]
vascularization	*in ovo*	CAM surface	4	metallic NP	[32]
	*ex ovo*	CAM surface	3	metallic NP	[33]
	*in ovo*	CAM surface	9	metallic NP	[34]
	*in ovo*	CAM surface	7 to 9	metallic NP	[35]
	*ex ovo*	CAM surface	7	silica NP	[36]
	*in ovo*	CAM surface	5	vincristine-loaded hydroxyapatite NP	[37]
	*in ovo*	CAM surface	4	carbon NP	[38]
	*in ovo*	CAM surface	6	surface engineered dendrimers	[39]
	*in ovo*	CAM surface	5	copolymeric NP	[40]
vascularization, lethality	*in ovo*	CAM surface	3	polysaccharide-doxorubicin-peptide bioconjugates “core-shell soft nanoparticles”	[41]
vascularization, CAM morphology	*in ovo*	CAM surface	7	betulin nanoemulsion	[42]
vascularization, chick morphology	*in ovo*	CAM surface	8	metallic NP	[43]
vascularization, femur ossification	*in ovo*	“injection” ^1^	7	metallic NP	[44]
vascularization, cell proliferation and tissue reaction	*in ovo*	CAM surface	8	metallic NP	[45]
CAM damage	*in ovo*	CAM surface	8	metallic NP	[46]
CAM damage	*in ovo*	CAM surface	7	lipid NP	[47]
CAM damage, lethality	*in ovo*	CAM surface	6	polymeric NP	[48]
CAM damage, lethality	*ex ovo*	intravascular	4	silica NP	[49]
CAM damage, lethality	*ex ovo*	intravascular	4	metallic NP	[50]
organ damage, organ distribution, lethality, tumor reduction	*in ovo*	intravascular	13	silica NP	[51]
morphological effect; embryo lethality	*ex ovo*	CAM surface	10	metallic NP	[52]
chick damage, microvascular injuries	*in ovo*	injection into the CAM´s mesoderm	11	lipodendriplexes	[53]
viability, biodistribution	*in ovo*	injection into the heart	3	alkylglyceryl-dextran-graft-poly(lactic acid) NP	[54]
vitality, lethality, embryo weight, iron content liver and kidney	*in ovo* (turkey!)	CAM surface	12	metallic NP	[55]
lethality and vein network degeneration	*in ovo*	CAM surface or intravascular	11	carbon NP	[56]

^1^ “Injection” was not further specified.

## Supplementary Materials

The following are available online at https://www.mdpi.com/2079-4991/10/12/2328/s1.
