# Supplementary material for: The Chorioallantoic Membrane Assay in Nanotoxicological Research—An Alternative for In Vivo Experimentation
Source: Nanomaterials (Basel). 2020 Nov 24;10(12):2328. doi: 10.3390/nano10122328 (PMC7760845; doi:10.3390/nano10122328)
Supplement: Supplementary file 1 [file nanomaterials-10-02328-s001.pdf]

PRISMA 2009 Flow Diagram

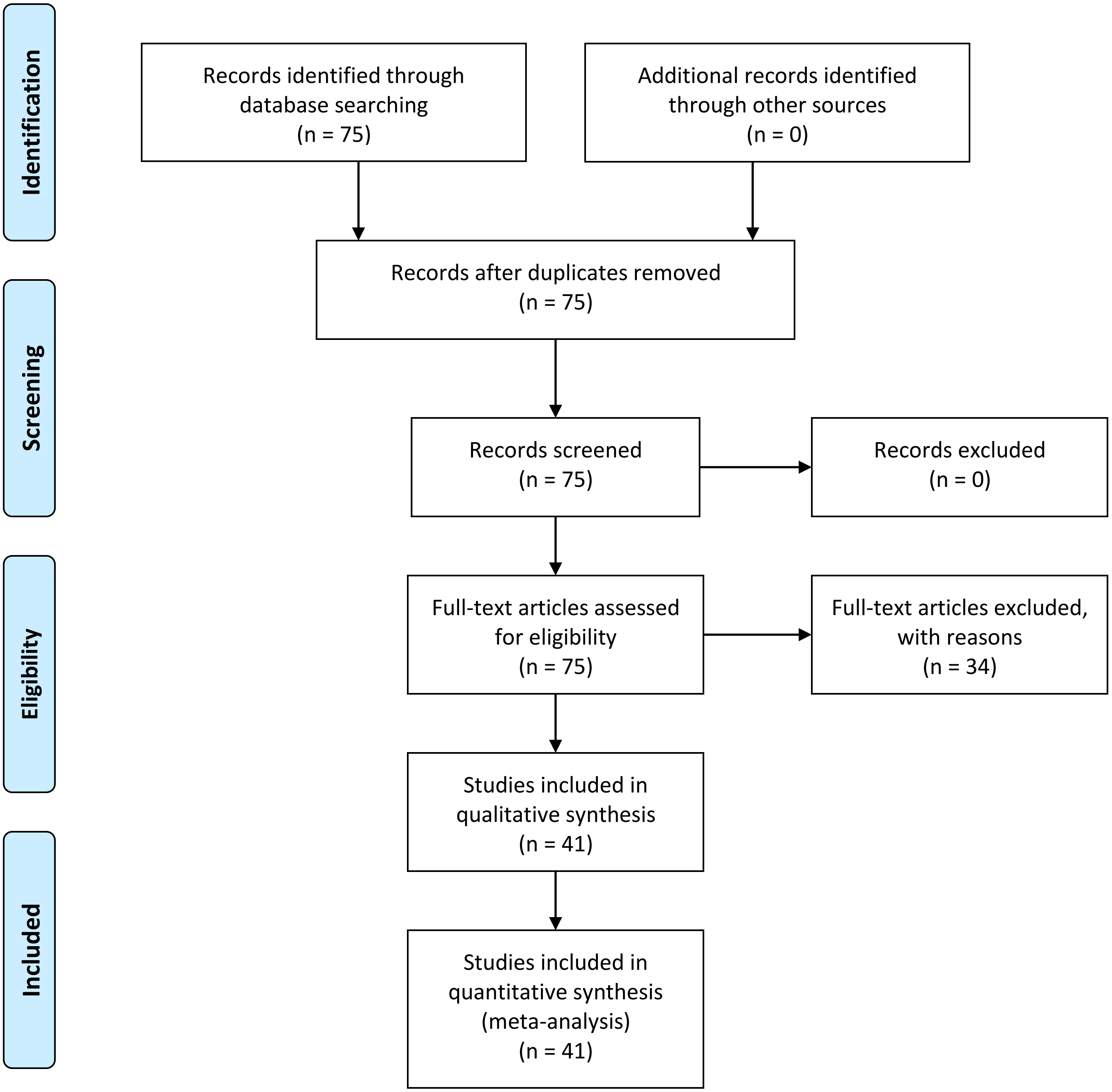

From: Moher D, Liberati A, Tetzlaff J, Altman DG, The PRISMA Group (2009). *P*referred *R*eporting *I*tems for *S*ystematic Reviews and *M*eta-*A*nalyses: The PRISMA Statement. PLoS Med 6(7): e1000097. doi:10.1371/journal.pmed1000097

For more information, visit www.prisma-statement.org.
